# Multilevel selection theory informs context-dependent mycorrhizal functioning

**DOI:** 10.3389/frmbi.2025.1676639

**Published:** 2026-01-05

**Authors:** Anne M. Katula, Nancy Collins Johnson, V. Bala Chaudhary, Michelle E. Afkhami

**Affiliations:** 1 Department of Biology, University of Miami, Coral Gables, FL, United States; 2 Biogeochemical Sciences Branch, Cold Regions Research and Engineering Laboratory, Hanover, NH, United States; 3 Department of Biological Sciences, Northern Arizona University, Flagstaff, AZ, United States; 4 School of Earth and Sustainability, Northern Arizona University, Flagstaff, AZ, United States; 5 Department of Environmental Studies, Dartmouth College, Hanover, NH, United States

**Keywords:** multilevel selection theory, arbuscular mycorrhizal fungi, holobiont, cooperation, context-dependency

## Abstract

Arbuscular mycorrhizal (AM) fungi form widespread, ancient, and critically important symbioses with plants, but their functioning and beneficial effects are highly context-dependent. This variability stems from eco-evolutionary dynamics operating across multiple levels of biological organization (e.g., genes to holobionts), making generalizable predictions about mycorrhizal outcomes challenging. Multilevel selection theory (MLST), which posits that selection acts simultaneously on multiple levels of biological organization including in opposite directions, can serve as a powerful framework for interpreting this variability in mycorrhizal functional phenotypes. Here, we outline the key principles of MLST and explore how its application to AM fungal symbioses can improve our understanding of this ubiquitous symbiosis. We highlight how four levels of biological organization important to AM symbioses – genes, nuclei, spores, and holobionts – can serve as one or more units of selection under a tripartite framework for the units of selection. We then examine how ecological contexts, such as stress, spatial structure, and community composition, can modulate the balance of selective forces across levels, ultimately shaping the degree of cooperation among symbiotic partners. We conclude by proposing future research directions using MLST to generate deeper insights into the complexity and adaptability of this globally important symbiosis.

## Introduction

Arbuscular mycorrhizal (AM) fungi are an ancient and widespread lineage of soil-borne microbes that form symbiotic associations with over 80% of terrestrial plant species (Van Der Heijden et al., 2008; Tedersoo et al., 2014). These interactions are often mutually beneficial (i.e., mutualistic), with AM fungi facilitating crucial plant functions such as nutrient acquisition (Rouphael et al., 2015) and stress tolerance (Ruiz-Lozano, 2003; Abdel-Salam et al., 2018) in exchange for photosynthetically derived carbon. The nature of this interaction, however, is context-dependent and can shift along a continuum from mutualism to parasitism under different biotic and abiotic contexts, creating enormous variability in mycorrhizal functioning (Johnson et al., 1997; Hoeksema et al., 2010; Bennett and Groten, 2022).

The context-dependency of AM symbioses results from a complex interplay of ecological (Antunes et al., 2025) and evolutionary (Hoeksema et al., 2018) forces that make it challenging to create generalizable predictions about mycorrhizal outcomes. Interpreting the variability in costs and benefits of this widespread symbiosis requires understanding how ecological interactions and evolutionary processes jointly shape symbiotic phenotypes, including emergent traits that are a product of the symbiosis rather than either partner individually (Johnson and Gibson, 2021). Therefore, describing mycorrhizal phenotypes requires consideration of multiple entities not only across different conditions (Johnson et al., 1997; Hoeksema et al., 2010; Bennett and Groten, 2022) but also across different levels of biological organization. It is important to distinguish between mycorrhizal fungi (the fungal organism) and the mycorrhiza (the symbiotic interaction) and clarify that functional outcomes are not intrinsic properties of the fungi but rather emergent traits of their interactions (**Box 1**). Because AM fungi are obligate symbionts, the mycorrhizal phenotype or “functioning” is often measured in terms of benefits conferred to plant fitness (e.g., mycorrhizas are considered mutualistic when net benefits to plants exceed net costs; Johnson et al., 1997). Importantly, we are still describing the outcome of the higher-level interaction. The evolution of the mycorrhiza, therefore, is fundamentally dependent on at least two entities, and driven by the tension between the cooperative traits that benefit all entities and the selfish traits that benefit constituent individuals (Bahar, 2018; Johnson and Gibson, 2021).

Box 1 **Glossary of terms.**

  • Arbuscular mycorrhizal (AM) fungi - Soil-borne microbes belonging to the subphylum Glomeromycotina (Spatafora et al., 2016) that form symbiotic associations    with plants. When using terms “AM fungi” or “mycorrhizal fungi” we refer to the fungal organism, not the association with plants or bacterial symbionts.

• Biological market models - Describe mutualisms as systems where organisms exchange resources or services based on partner choice or supply-and-demand    dynamics (Noë and Hammerstein, 1994)

• Context-dependency - When functional outcomes vary with environmental or ecological conditions (Bronstein, 1994)

• Holobionts - Integrated multispecies consortia interacting at different developmental, ecological, and evolutionary scales simultaneously (Chiu and Gilbert, 2015)

• Interactors - Entities that engage with the environment through their traits such that there is differential replication/reproduction/reconstitution   (Suárez and Lloyd, 2023)

• Manifestors of adaptation - Entities which reflect the accumulated modification of traits resulting from a history of selection acting on interactors   (Suárez and Lloyd, 2023)

• Multilevel selection theory - posits that selection can occur simultaneously across two or more levels of biological organization, and that the direction of selection at   1these levels can be in opposition (Suárez and Lloyd, 2023)

• Mutualism - Interspecies interactions characterized by positive effects on survival and/or reproduction for all participants (Holland and Bronstein, 2008)

• Mycorrhiza - Traditionally describes the symbiotic interaction between a mycorrhizal fungus and a plant but may be extended to include hyphosphere-  associated bacteria

• Mycorrhizal functioning/phenotype - Often used to describe the outcome of the mycorrhizal interaction along the mutualism-parasitism continuum   (Johnson et al., 1997)

• Non-genetic traits - Traits that result from non-genetic (e.g., epigenetic or cytoplasmic) modes of inheritance

• Parasitism - Interspecies interactions in which a symbiont (i.e. the parasite) exploits resources from the host with negative consequences for host fitness   (Combes, 2004)

• Replicators/reproducers/reconstitutors - Entities responsible for heritability through copying (replicators), copying and development (reproducers), or non-genetic   recreation (reconstitutors) (Suárez and Lloyd, 2023)

• Stability of Traits - Repeated reappearance of phenotypic variants across generations, even without lineage-based inheritance (Suárez, 2020)

• Transposable elements - DNA sequences that can move to different sections within a genome (Oliveira et al., 2025)

• Tinkering/engineering adaptations - Traits that result from a history of selection acting at a specific level and *appear* as though they were ‘designed’ to fit or respond   to specific environmental challenges (Suárez and Lloyd, 2023)

Prevailing evolutionary frameworks for studying such cases of biological cooperation, such as biological market models, have greatly advanced our understanding of AM fungal systems by conceptualizing mutualisms as dynamic exchanges governed by supply and demand (Noë and Hammerstein, 1994). In the context of the mycorrhizal symbiosis, biological market models posit that plants and fungi act as trading partners, exchanging carbon and nutrients at context-dependent exchange rates. This framework has been used effectively to explain why plants should allocate more carbon to fungi that provide more phosphorus, thereby promoting cooperative interactions in some contexts (Schwartz and Hoeksema, 1998; Kiers and Denison, 2008). However, biological market models typically assume discrete trading agents, which AM fungi complicate (Noë and Kiers, 2018). For example, their spores are multinucleate and form aseptate and genetically-heterogeneous (Kokkoris et al., 2020) networks that are capable of fusing with others (Giovannetti et al., 2015), leading to unclear genetic boundaries. Additionally, unlike all other known eukaryotes, AM fungi may not experience a single-nucleus bottleneck (Marleau et al., 2011), which Dawkins (1976) argues are fundamental to the formation of discrete organisms as they promote genetic cohesion. This biological entanglement indicates that models which rely heavily on the concept of individuality may not provide a complete framework for studying AM fungi and their symbioses, and may benefit from a complementary approach that can accommodate multiple, potentially conflicting, levels of selection.

We propose that multilevel selection theory (MLST) provides a powerful conceptual model for addressing context-dependent functioning to achieve important insights and a more sophisticated understanding of the evolution of mycorrhizal symbioses. Multilevel selection theory posits that selection can operate on multiple levels of biological organization simultaneously and, importantly, that the direction of selection at these levels may be in conflict (Okasha, 2006; Wilson and Wilson, 2007). Therefore, MLST offers a flexible paradigm for interpreting variability in mycorrhizal functioning by clarifying the evolutionary mechanisms that underlie divergent phenotypes. In this perspective, we present a framework for understanding how multilevel selection influences the evolutionary trajectories of symbiotic stability and function, emphasizing the role of opposing selection across different levels of biological organization. By informing context-dependent mycorrhizal functional outcomes, integrating an MLST perspective into prevailing frameworks is critical for improving mycorrhizal management in sustainable agriculture and ecosystem restoration.

## Multilevel selection theory

The origins of MLST can be traced back to Charles Darwin. Although his work was clearly focused on individual selection, where traits that increase an individual’s survival and reproductive success are favored, he invoked something akin to group selection to explain the evolution of altruism and cooperation in humans (Darwin, 1871; Borrello, 2005). Consequently, in the early 20^th^ century, group selection became popular for explaining how morphological traits and behaviors may evolve for the benefit of the community or species. However, by the mid-20^th^ century, the synthesis of Darwinian theory with Mendelian genetics pushed evolutionary thinking towards gene-centrism, and group selection became unpopular (Borrello, 2005). Today, there is a growing consensus that group selection may in fact be informative under the broader scope of MLST (Traulsen and Nowak, 2006; Pievani, 2014; Kramer and Meunier, 2016; Bourrat, 2025). MLST synthesizes individual and group selection, offering greater flexibility for interpreting evolutionary processes as a function of ecological context (Kniffin, 2021). MLST, though originally applied to individuals and single-species groups, has been extended to explain functional organization at higher levels, including multispecies communities (Wilson, 1997), or lower levels including genes (Dawkins, 1976), and even nuclei (Jany and Pawlowska, 2010; Manyara et al., 2024).

**Figure 1:**
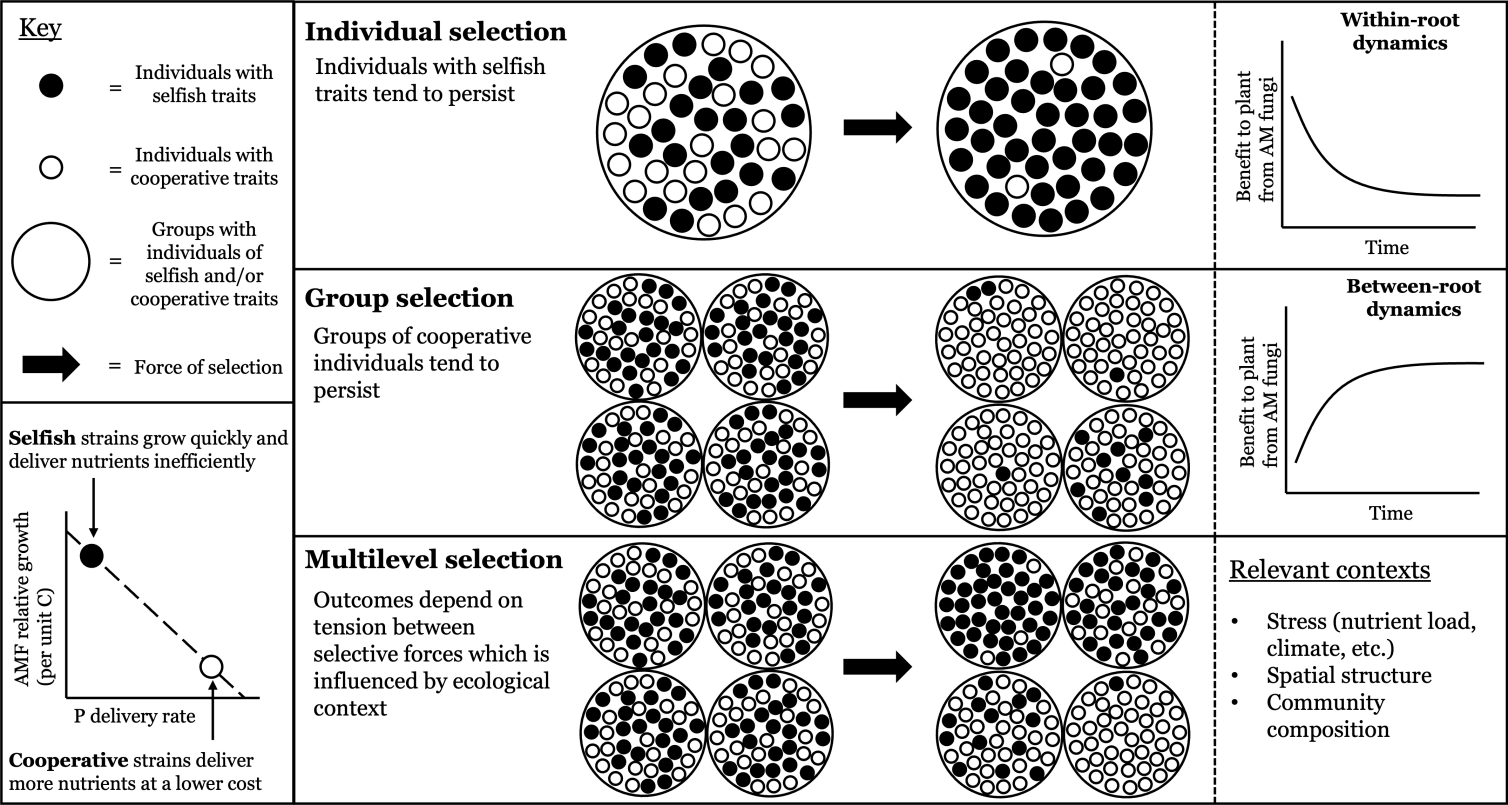
Selection may operate on multiple levels of biological organization resulting in different phenotypic outcomes within and between groups. Selection on the level of the individual will tend to produce groups of individuals with selfish traits (black circles), as the process optimizes individual fitness. This occurs in AM fungal systems where strains are homogenized such that they are intermixed on a single root, resulting in the accumulation of selfish (i.e., exploitative) fungi and a net decline in the benefit of AM fungi to the plant over time (Bever, 2024). Alternatively, selection on the group level will tend to produce groups consisting of individuals with cooperative traits (white circles), as the process optimizes group fitness. This occurs in AM fungal systems where the strains are spatially structured such that they are on different roots, resulting in the accumulation of cooperative fungi and a net increase in the benefit of AM fungi to the plant over time (Bever, 2024). Under multilevel selection, selection at both levels occurs simultaneously. Resulting phenotypes (selfish or cooperative traits) depend on the balance between these selective forces, which is influenced by ecological contexts such as stress or spatial structure. This can result in groups with mostly selfish traits, mostly cooperative traits, or a combination of both, making it challenging to create generalizable predictions about phenotypic outcomes without considering ecological context and the mechanism underlying plant and mycorrhizal responses.

MLST is especially useful in systems where there is conflict between selection at different levels of organization (Luo, 2014). This conflict often results in trade-offs where individual selection favors competition (promoting individual fitness) while group selection favors cooperation (promoting collective fitness) (Griffing, 1967) (**Figure 1**). For example, in a study of *Impatiens capensis*, selection at the individual level favored larger plants for their competitive advantage in sequestering resources. However, at the group level, plants in groups of smaller average size had higher overall fitness because the resources were shared equally (Stevens et al., 1995). Similarly, in a human model of host-virus interactions, fast-replicating strains may exhibit a competitive advantage at the individual level by outcompeting slower replicating strains within the same host. However, if rapid viral replication increases host detection and immune response, thereby reducing the overall fitness of the viral population, fast replication may be selected against (Orlando et al., 2012). The tension between immediate local-scale benefits and broader ecological costs illustrates the central concept of MLST: opposing selection pressures operating simultaneously at different biological scales.

## Disambiguating the units of selection

**Figure 2:**
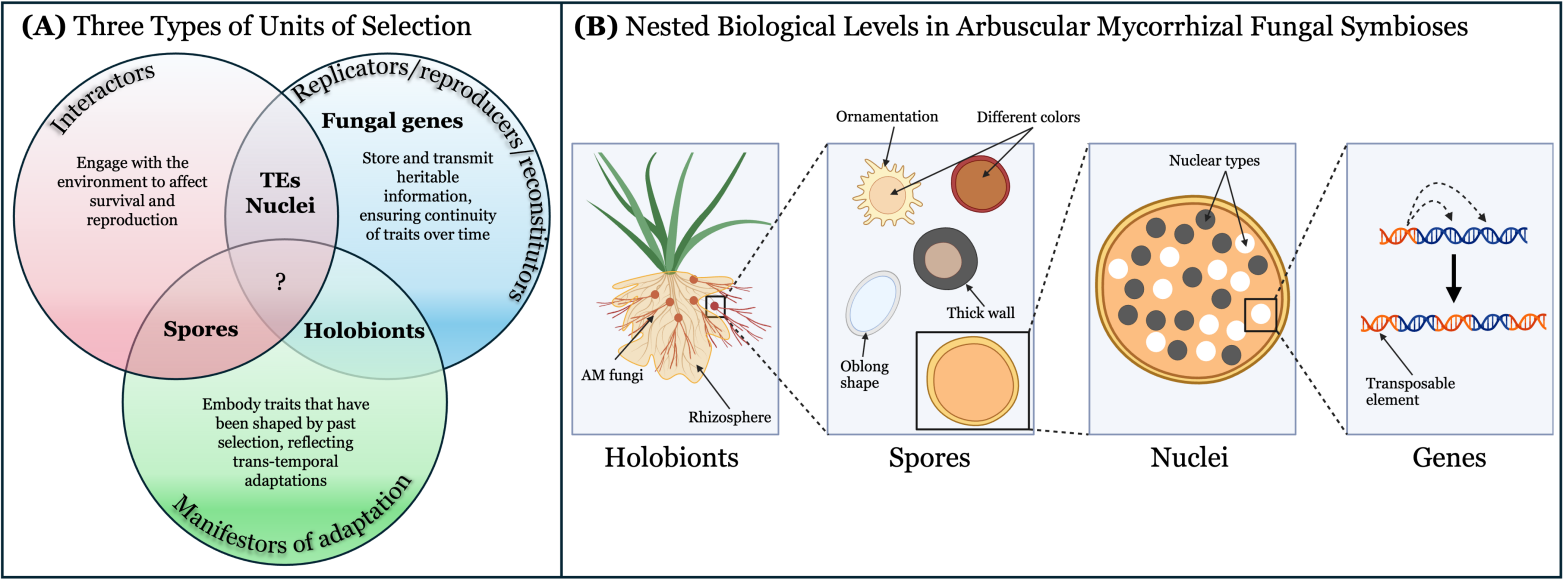
**(A)** Biological levels may fulfill one or more roles of the three units of selection, including interactors, replicators/reproducers/reconstitutors, and manifestors of adaptation. The functional roles of the three units are distinct and non-coextensional, meaning a single biological level may satisfy multiple but does not necessarily need to. It is unclear if there is any biological entity in the AM fungal life history which fulfills all three functional roles. **(B)** In AM fungi, candidate levels of selection are hierarchically nested and may consist of fungal genes (including transposable elements, which are a special case), nuclei, spores, and holobionts. Created in BioRender. Afkhami, M. (2025) https://BioRender.com/kg4zkcz.

Which biological entities can evolve by means of natural selection has been the primary concern of the units or levels-of-selection debates (Suárez and Lloyd, 2023). Importantly, different meanings of the expression “units of selection” underlie much of the conflict in such debates. Suarez and Lloyd postulate that there are at least three types of units of selection, each encompassing a distinct functional role that biological entities might fulfill in the process (2023). Disentangling these roles is necessary for understanding how multilevel selection shapes complex ecosystems (**Figure 2**).

Suárez and Lloyd (2023) propose three types of units of selection: (1) replicators/reproducers/reconstitutors, (2) interactors, and (3) manifestors of adaptation. The first type of unit of selection includes replicators, reproducers, and reconstitutors, which are all entities responsible for heritability. However, they each fulfill this role through distinct mechanisms. Specifically, they are “differentially copied (replicators), differentially transmitted through material overlap (reproducers), or differentially recreated in the absence of copy or material overlap (reconstitutors) across generations” (Suárez and Lloyd, 2023, p. 6). High copy fidelity is central to the replicator concept, with genes being the canonical example (Dawkins, 1976; Hull, 1980). Reproducers are entities that engage in the process of reproduction, whereby material propagules (genetic or otherwise) multiply and confer the capacity to develop (Griesemer and Wade, 2000). Unlike replicators, high copy fidelity is not emphasized for reproducers, accommodating mechanisms of both genetic and non-genetic (e.g. epigenetic or cytoplasmic) inheritance (Adrian-Kalchhauser et al., 2020). In this view, replicators may be considered a highly specialized class of reproducer (Szathmáry and Smith, 1995). Reconstitutors, on the other hand, are units that recreate or re-establish traits across generations through alternative inheritance mechanisms regardless of the biomolecular basis (Veigl et al., 2022). They enable the Stability of Traits concept, which refers to the repeated reappearance of phenotypic variants across generations, even without lineage-based inheritance (Suárez, 2020; Bapteste and Papale, 2021; Veigl et al., 2022). For example, holobionts (host-microbiome multispecies consortia), such as vampire bats and their gut microbiota, are reconstitutors for traits like sanguivory (Suárez and Lloyd, 2023). Sanguivory is transgenerationally recreated and maintained as a stable trait via the assembly of many independent bacterial lineages, most of which come from the environment (Veigl et al., 2022). The second type of unit of selection includes interactors, which are entities that interact with the environment through their traits in a way that causes differences in replication, reproduction, or reconstitution. The third type of unit of selection includes manifestors of adaptation, which are entities that possess tinkering/engineering adaptations as a result of selection processes, reflecting accumulated modifications of traits, often in the form of highly specialized biological function. Importantly, these units are “non-co-extensional,” meaning they need not correspond to the same biological entity or level within the biological hierarchy (Suárez and Lloyd, 2023). That is, while a single entity may fulfill all of these roles, there is no reason they must (Marín, 2024). Differentiating between these functional roles enables a clearer understanding of how selection can act on multiple levels of organization simultaneously (Johnson and Gibson, 2021; Marín, 2024).

## Applying multilevel selection theory to AMF

### What levels does selection act on in AMF?

#### Fungal genes

The concept of gene-level selection, popularized by Richard Dawkins (1976), has sparked considerable debate in evolutionary biology. In his foundational work, Dawkins proposed that genes are functionally immortal, acting as the primary units and ultimate beneficiaries of natural selection. However, opponents of genic selection maintain that while genes are crucial, natural selection ultimately operates on phenotypes through interactions with the environment (Mitton, 1987; Marín and Wade, 2025). This is clarified by distinguishing between the functional roles of different units of selection; as entities that are differentially copied across generations, genes clearly function as replicators regardless of the extent that they interact with their environment (Suárez and Lloyd, 2023). However, in some cases, such as with selfish genetic elements, genes may function as both replicators and interactors. Selfish genetic elements are stretches of DNA that can enhance their own transmission at the expense of other genes in the genome, even if it results in fitness costs for the organism (Ågren, 2016). For example, transposable elements can self-replicate throughout the genome with variable consequences for the functioning of other genes and the host (Arber, 2000). This may particularly apply to the AM symbiosis because AM fungi contain large genomes rich in transposable elements. Importantly, these elements are positioned near genes involved in host interactions and gene regulation, indicating evolutionary and functional relevance for the arbuscular mycorrhiza (Oliveira et al., 2025).

#### Nuclei

AM fungi form networks that are coenocytic (i.e. multinucleate) and aseptate (i.e. lacking compartmentalization), allowing thousands of free-flowing nuclei in shared cytoplasm to move across hyphal networks. Jany and Pawlowska (2010) investigated nuclear dynamics during spore formation and found that nuclei within the hyphae showed asynchronous replication, mobility, and degradation, supporting the presence of selection at the level of individual nuclei. Manyara et al. (2024) investigated intra- and inter-organismal genetic variation of AM fungi by analyzing single nuclei from three strains of two Claroideoglomus species. They observed a low dN/dS ratio (non-synonymous to synonymous mutations), which is a strong signature of purifying selection at the level of the nuclei. As entities that demonstrate variation and differential fitness (but do not exhibit traits reflective of biological tinkering) nuclei may fulfill the function of interactors. Additionally, it is possible that nuclei simultaneously function as replicators. Dawkins specifies that an organism is not a replicator, but its genome may be if it is asexually reproduced (1976), which is true of AM fungi. Given that AM fungal networks are genetically heterogeneous, it may be most appropriate to consider a ‘genome’ as that which is contained within a single nuclear package.

#### Spores and hyphae

AM fungi form soil-borne spores that provide numerous ecological functions, including dispersal and persistence under stressful conditions or in the absence of appropriate plant partners. Spores vary with respect to quantifiable and heritable (Bever and Morton, 1999) morphological traits, such as size, color, shape, ornamentation, and wall thickness (Chaudhary et al., 2025). These traits directly interact with the environment differentially impacting colonization and subsequent reproduction. For example, spore size influences dispersal (Chaudhary et al., 2020), and spore color is important for spore defense and persistence (Hopkins and Bennett, 2023). Following spore germination and host colonization, AM fungi form extensive mycorrhizal networks comprising interconnected hyphae that mediate crucial functions including nutrient exchange between fungi and hosts, resource exploration and acquisition (Smith and Read, 2008), and recruitment of bacterial associates (Jiang et al., 2021, Wang et al., 2023). Hyphae interact with hosts and the environment through traits related to morphology, fluid mechanics, and network architecture, directly impacting fungal persistence and reproduction (Antunes, 2025). For example, volumetric flow rate and velocity influence nutrient transport efficiency, and network connectivity influences resource redistribution and resilience to damage or disturbance (Antunes, 2025). Therefore, both spores and hyphae act as interactors under Suarez and Lloyd’s tripartite framework (2023). It is challenging to distinguish if spores and hyphae also act as manifestors of adaptation, reflecting a history of biological tinkering. One reason for this is that the fitness effects of many of these traits have not been tested empirically, and their operational significance remains speculative. Interestingly, a recently constructed phylogeny revealed that spore ornamentation is not significantly conserved among AM fungi despite high incidence across mycorrhizal taxa (Cooper, 2023). This could suggest that ornamentation, having convergently evolved across AM fungi, is an adaptive trait and reflects a history of biological tinkering. As such, spores may function as manifestors of adaptation with respect to some traits, reflecting a history of adaptation resulting from biological tinkering, but further research is needed to determine the extent to which this is true of hyphal traits.

#### Holobionts

The holobiont concept offers a theoretical framework for studying the interactions between hosts and their associated microbes. This perspective treats the host-microbe system as a selectable unit, where the combined fitness of the host and its microbiota is shaped by their interactions. Some argue that holobionts cannot be units of selection because they reproduce independently. However, for some types of units of selection, namely manifestors, mode of inheritance is irrelevant, and the emphasis is instead placed on cooperation among entities. According to Stencel and Wloch-Salamon (2018, p. 201; reviewed in Suárez and Lloyd, 2023), two units may evolve a high degree of interdependence, communication, and morphological integration, thus acting as manifestors, but might reproduce independently, thus not being replicators/reproducers. In the case of the arbuscular mycorrhiza, there are clearly sophisticated mechanisms of interaction, including specialized morphological structures (Antunes et al., 2025) that facilitate dynamic resource exchange (Noë and Kiers, 2018) and complex chemical signaling (reviewed in Wang et al., 2017), suggesting they may function as manifestors of adaptation. It is also possible that the mycorrhizal holobiont could function as a reconstitutor for traits like organic phosphorus solubilization. Even though P uptake is one of the most important functions AM fungi provide plants, they actually have a limited capacity to mineralize organic phosphorus (Tisserant et al., 2013) and may outsource this capability to bacterial symbionts, which they can recruit to the hyphosphere via exudation (Qin et al., 2016). In a recent study, Wang et al. (2023) showed that AM fungi assemble a distinct, core hyphosphere microbiome with functional significance in organic P solubilization. These findings suggest that traits like P mineralization are not performed by AM fungi alone but are reconstituted through the recurring assembly of functionally relevant microbial partners. Even if the specific taxa vary, the consistent reappearance of this trait points to the Stability of Traits concept, supporting that the multipartite interaction between plants, fungi, and their microbial associates may function as a reconstitutor. Importantly, however, the holobiont concept is not always appropriate for the mycorrhizal symbiosis, where selection at lower levels can lead to decoupling. But under the MLST framework, the holobiont concept can be applied with more flexibility and does not need to be applicable in every context.

## Case study and future directions

Theory predicts that the evolution of cooperation is unstable in the absence of mechanisms that restrain exploitation (Trivers, 1971; Bull and Rice, 1991; Frederickson, 2013). Yet, in AM fungal systems, there is persistent variation in symbiont quality. MLST can explain the maintenance of this variability, as demonstrated by studies with C*laroideoglomus candidum* (formerly named *Glomus candidum*) and *Gigaspora margarita* which illustrate how selection can act in opposing directions across levels in AM fungal systems simultaneously (Bever et al., 2009; Ji and Bever, 2016). In a greenhouse experiment, *C. candidum* behaved cooperatively by delivering phosphorus to the plant efficiently while maintaining a relatively low growth rate per unit of carbon; in contrast, *G. margarita* behaved exploitatively, growing rapidly but delivering phosphorus inefficiently (Ji and Bever, 2016) (**Figure 1**). In models focused on root-level dynamics, exploitative strains have a competitive advantage because the delivery of benefits to symbiotic partners is costly (Bronstein, 2001; Bever, 2002), suggesting selection at the “individual” level (that is, between fungal strains) favors selfishness (e.g., rapid growth). However, split-root experiments revealed that plants preferentially allocated carbon to *C. candidum* relative to *G. margarita* (Bever et al., 2009). This is consistent with results from axenic culture experiments using several Glomus species that demonstrate hosts select for more beneficial AM fungi (Hammer et al., 2011; Kiers et al., 2011). These findings illustrate that partner choice mechanisms enable selection for cooperative traits at the holobiont level by stabilizing mutualisms with beneficial partners despite their competitive disadvantage. Importantly, selection for cooperative *C. candidum* was only observed when the fungal strains were spatially structured into distinct patches, such that the plant possessed the spatial resolution to effectively promote more beneficial taxa. However, selection for exploitative *G. margarita* was observed when spatial structure was low because the plants were unable to discriminate between strains (Bever et al., 2009). In the absence of higher-level selective control due to ecological context (e.g., spatial structure and community composition), selection at the “individual” level dominated. Overall, this example illustrates that MLST can elucidate important insights, including how ecological context shapes the evolution of cooperation and exploitation in symbiotic interactions. Furthermore, it suggests that MLST can provide a strong predictive framework for understanding the eco-evolutionary dynamics of mycorrhizal symbioses.

Consideration of MLST also spotlights important new research directions to improve our understanding the ecological and evolutionary trajectories of mycorrhizal symbioses. For example, *how often are different levels of selection aligned versus in conflict, and what factors influence this balance?* Understanding the frequency and drivers of alignment versus conflict across levels can reveal how often ecological contexts constrain or mask evolutionary outcomes. More insight into when and why trade-offs occur could strengthen our predictions of mycorrhizal phenotypes, which will be especially important in the context of anthropogenic global change. In addition, it would be valuable to investigate *how stable are reconstituted traits across multiple generations?* Interrogating the stability of non-genetic traits can clarify the mechanisms that enable functional persistence in the absence of genetic continuity. This has implications for understanding the evolution of emergent traits and their potential resilience in symbiotic systems. Finally, *how does anthropogenic stress modulate selection across levels?* Global change factors such as warming, nutrient fertilization, and land use change may shift the balance of selection between levels. For example, as global phosphorus inputs increase (Nesme et al., 2018), selection at the holobiont level may be undermined and selection between competitive fungal strains promoted. This could explain why environments with luxury soil resources often favor neutral or even antagonistic microbes in the rhizosphere (Johnson, 1993; Lekberg et al., 2021; Johnson and Marín, 2025). Investigating such questions could support a mechanistic understanding of how MLST shapes mycorrhizal symbioses in real ecological contexts, which may help resolve persistent challenges in mutualism theory (e.g., *why not only associate with the best mutualist*)*?*. Additionally, this work may provide a template for using MLST to accommodate the complex biology of other symbiotic systems into selection theory (e.g., horizontal gene transfer in rhizosphere bacteria; Andrews et al., 2018; Ghaly et al., 2024; Cotta et al., 2025). In the long term, theories like MLST which embrace the inherent multiscale nature of these widespread symbioses can enhance efforts to harness natural species interactions in soil restoration and sustainable agriculture.

## Conclusion

Multilevel selection theory, in tandem with existing frameworks for biological cooperation, offers a flexible approach for understanding context-dependence in mycorrhizal functioning. As AM fungi engage in complex interactions with plants, these partnerships can shift along the mutualism-parasitism continuum based on a variety of biotic and abiotic factors. MLST clarifies that selection pressures act simultaneously at multiple levels, from genes and nuclei to individuals and communities, and that evolutionary outcomes are driven by ecological context. MLST provides a paradigm for interpreting this interplay of ecological and evolutionary forces to guide us towards a more comprehensive understanding of the dynamic, context-dependent nature of mycorrhizal interactions.

## Data Availability

The original contributions presented in the study are included in the article/supplementary material. Further inquiries can be directed to the corresponding author.
